# Design and Implementation of an Open-Source SCADA System for a Community Solar-Powered Reverse Osmosis System

**DOI:** 10.3390/s22249631

**Published:** 2022-12-08

**Authors:** Sheikh Usman Uddin, Mirza Jabbar Aziz Baig, Mohammad Tariq Iqbal

**Affiliations:** Department of Electrical and Computer Engineering, Memorial University of Newfoundland, 230 Elizabeth Ave, St. John’s, NL A1C 5S7, Canada

**Keywords:** SCADA, Node-Red, reverse osmosis, Grafana, solar energy, open-source

## Abstract

Design and implementation of an open-source-based supervisory control and data acquisition (SCADA) system for a community solar-powered reverse osmosis are presented in this paper. A typical SCADA system available on the market is proprietary and has a high initial and maintenance cost. Aside from that, there is no SCADA system with an alert system available to give users updates and status information concerning the system. The objective of this study is to develop a comprehensive SCADA design that takes advantage of open-source technology to address the world’s most pressing problem, access to clean water. The designed reverse Osmosis system also uses renewable energy-based power sources. In this system, all data is stored and analyzed locally, which ensures the data is secure and allows the user to make data-driven decisions based on the collected data. Among the main components of this system are the field instrument devices (FIDs), the remote terminal unit (RTU), the main terminal units (MTUs), the web-based programming software, and the data analytics software. The Node-Red programming and dashboard tool, Grafana for data analytics, and InfluxDB for database management run on the main terminal unit having Debian operating system. Data is transmitted from the FIDs to the RTU, which then redirects it to the MTU via serial communication. Node-Red displays the data processed by the MTU on its dashboard as well, as the data is stored locally on the MTU and is displayed by means of Grafana, which is also installed on the same MTU. Through the Node-Red dashboard, the system is controlled, and notifications are sent to the community.

## 1. Introduction

A SCADA integrates software and hardware to enable monitoring and control of any industrial process through sensors and control algorithms [1]. SCADA enables the process to be better controlled, and decisions can be made based on data analytics. In SCADA, sensors feed data to control systems through hardware components. The control system is used to process all the data and display it using a Human Machine Interface (HMI). A SCADA can record all events in a database. Additionally, the system includes a reporting system to ensure that alerts and status messages are delivered to users. A SCADA system minimizes downtime, increases system availability, and allows organizations to make more informed decisions with less effort. Among the most critical components of a SCADA system is an RTU or programmable logic controller (PLC). During operation, the PLC or RTU communicates with the centralized computer, which contains SCADA software attached to a database containing historical data information. Field devices provide data to the PLC or RTU, which processes the data to the SCADA software and implements important decisions [1,2].

Approximately 70% of the earth is contained in water. However, only 3% of it is fresh water, the remainder being frozen glaciers or unusable. Around 1.1 billion people globally don’t have access to water, and almost 2.7 billion don’t have water availability for at least one month in a year [3]. One of the biggest challenges that the world is facing is a huge water crisis worldwide, and countries are looking for different ways in order to extract water. Desalination of water is one of the solutions available today for the production of clean water, but this process requires a huge amount of energy which in turn is achieved by burning fossil fuels. This contributes to global warming, which is another biggest concern. Considering the excessively increasing requirement for water, various renewable energy-based reverse osmosis desalination systems could provide a great source of clean and fresh water. As the water passes through membranes, solar-powered reverse osmosis converts brackish water to fresh water, removing approximately 98% of the salt from the water [4]. The complete system will be powered by photovoltaic (PV) panels that convert solar energy to the form of electrical energy. The electrical energy is then further utilized to power the reverse osmosis-based desalination system. Therefore, after completing the sizing [5] and dynamic modeling using the bond graph method of the system [6], the SCADA system is designed for real-time monitoring that enables local data logging embedded with a global system for mobile communication (GSM) based short message service (SMS) alerts system. The SMS alert system will provide all live updates to the community about the status of the system.

Throughout this article, information is organized in the following way. Section 2 of the paper presents a detailed literature review. Section 3 of this article describes the entire system in detail, while Section 4 describes the components in detail. This article presents a description of the implementation methodology in Section 5 and discusses the results of the implementation, as well as a design and discussion of the prototype in Section 6. In Section 7, a discussion is carried out, and the article concludes in Section 8.

## 2. Literature Review

Through the course of this study, a thorough literature review was performed, and a number of useful sources were identified and summarized in this section. In [7], a detailed SCADA/HMI was developed for a multi-stage desalination system. The desalination system consists of eight cycles with many field sensors. The control system used was Siemens S7-300 PLC with WINCC SCADA software which is a fee-based system. The authors highlighted the idea of having a redundant system in case the main server is failed. The authors further concluded that the multi-point interface (MPI) is used as the main control loop because it is faster than an Ethernet connection. In [8], authors have developed a personal computer-centered SCADA system for reverse osmosis desalination plants. The basic setup includes LabView Software which is fee-based, with a data acquisition card to build the complete setup. This system provides a low-cost SCADA system where basic functionality and control for small systems can be achieved. The authors of [9] worked on the monitoring and control of multi-stage flash brine recirculation (MSF-BR) combined with the reverse osmosis (RO) system. The system is elaborated on in detail, and its process control and instrumentation strategy are explained. They used a Rockwell Automation product called FactoryTalk View Site Edition (SE), which is a widely used software in the field of automation and control. The whole process control system was developed and controlled by the user-friendly HMI. In [10], the authors have elaborated on the controlling process by using distributed control systems (DCS), SCADA, and PLC. The research contribution educates the reader about the working of the DCS system with a focus on developing a control system for the reverse osmosis plant. The authors have developed an internal model-based control strategy to control the overall plant. The control strategy works on the multivariable control system to make the system control robust. Further, for database management, a text file is generated that keeps a record of all the alarms and sequences of events. The data history is also saved in the database as a text file. In [11], authors have designed and implemented SCADA for the huge desalination process where five lines of desalination run parallel in an integrated plant formed by merging four coastal wells and two end-of-line pumps of permeated water. The MOVICON 11.5 SCADA system is used as the software platform for the design. The work further explains the control system for each operation, followed by the centralized control and monitoring system. Each process has its own algorithms for the control system, and all individual algorithms were synchronized to achieve maximum efficiency. Extensive data set is available for the generation of alarms and reports and for creating a historical database. In addition, the paper provides trends and monitoring of the system using historical data to enable data-driven decision-making. All of the aforementioned examples use proprietary software and hardware.

A very large coastal desalination plant in California was retrofitted, and the authors [12] explained the work. Due to the increasing cost of electricity in California, the management decided to operate the plant just on weekends because the cost of electricity is lower on weekends resulting in less utilization of the plant compared to its full design potential. The retrofit project executed increased the operational efficiency of the system, which resulted in running the plant for all days of the week. Moreover, the plant’s operating costs were reduced by 64%, which was a significant achievement. The retrofit work includes modification of mechanical systems as well as elaborating future improvement plans of including SCADA systems for monitoring and predictive analysis. The research work further elaborated on the modular expansion of the system with a secured wireless SCADA network that will allow secure control and communication. In [13,14], authors have developed a control system for the reverse osmosis desalination process using PC based SCADA system. The authors [13] majorly focus on developing the fault tolerant based control system strategy, whereas the [14] system focuses on low-cost solutions with safety features. The detailed system architecture was discussed using Adam 4000 modules for hardware and VisiDaq 3.1 for software integration. Each integration provides easy to program solution with full control. Another research [15] has elaborated a descriptive mathematical model for a large-scale multi-scale flash (MSF) desalination process. The input is passed through a series of signal processing steps, including signal conditioning, filtering, steady state checks, and limit value monitoring. Extensive mathematical modeling is carried out to ensure that the process is optimized. In order to ensure system robustness, a fail-safe dual redundant computer system configuration in hot standby mode is discussed with a robust SCADA system having a self-system backup capability in case of system failure. These are some examples where authors used commercial hardware and software with no consideration of the cost and energy needed for operation.

A study conducted at the Massachusetts Institute of Technology demonstrated how a small solar-powered system produced clean water for a village community in Mexico [16] and was enough for the whole community. As part of another study, analysis was done to understand the behavior of the system according to the temperature of the solar panels. The study concluded that 10% more energy from the photovoltaic panels was generated when the system was cooled [17]. Another paper was reviewed in which elaborative instrumentation on just reverse osmosis system was discussed, and it concluded that the performance of such a system is highly dependent on the temperature and pH of the water [18]. Edward Fredkin and Roger Banks in [19] provide a new approach for reverse osmosis system instrumentation and control design by applying artificial intelligence-based software technology in order to make the system robust and more efficient. In [20], authors analyzed how the variation of renewable energy sources can affect the output of desalination water production. This work includes the parameter of flow and pressure. The authors of [21] worked on a reverse osmosis system without batteries. They further designed the instrumentation and data acquisition on LabView and analyzed the results of the system. The study utilizes the microprocessor-based programmable logic controller with active sensors to analyze the performance of an online reverse osmosis system plant [22]. The authors of [23,24,25,26] have also created designs utilizing open-source technology based on Arduino and MySQL as the database. Their work serves as a reference to the potential of creating a low-cost, open-source technology-based SCADA system but is limited in terms of execution and developing control and monitoring of reverse osmosis desalination system. Also, the author of [27] demonstrated the utilization of convolutional neural networks with the raspberry pi system in order to increase the real-time computational power of the system, whereas the authors of [28,29] have unique solutions and algorithms for providing cloud-based technology with advanced mobile networks for providing guidance on an efficient alert system to the community.

There have been several approaches explored by researchers to ensure the instrumentation and control of desalination systems without providing real-time alerts to users. Others have designed SCADA systems with multiple servers, which has compromised the security of the system as data is stored remotely. Thus, a comprehensive design and implementation of an open-source SCADA system are missing in the literature. In this paper, our focus is to design an open-source, low-cost SCADA system with the following features:

- With the latest SCADA architecture, this study stands out, particularly with respect to its use for reverse osmosis systems.
- The designed system is configured on a local machine through Node-Red visual programming language that is accessible through the browser for easy control.
- The system incorporates local storage with restricted user authorization only.
- Intuitive dashboards and data analytics are provided with a Web-based real-time and historian monitoring and control system.
- Maintains an alert system to inform the community of the system status and updates.
- The designed system uses 100% open-source technology.

## 3. System Description

**Figure 1:**
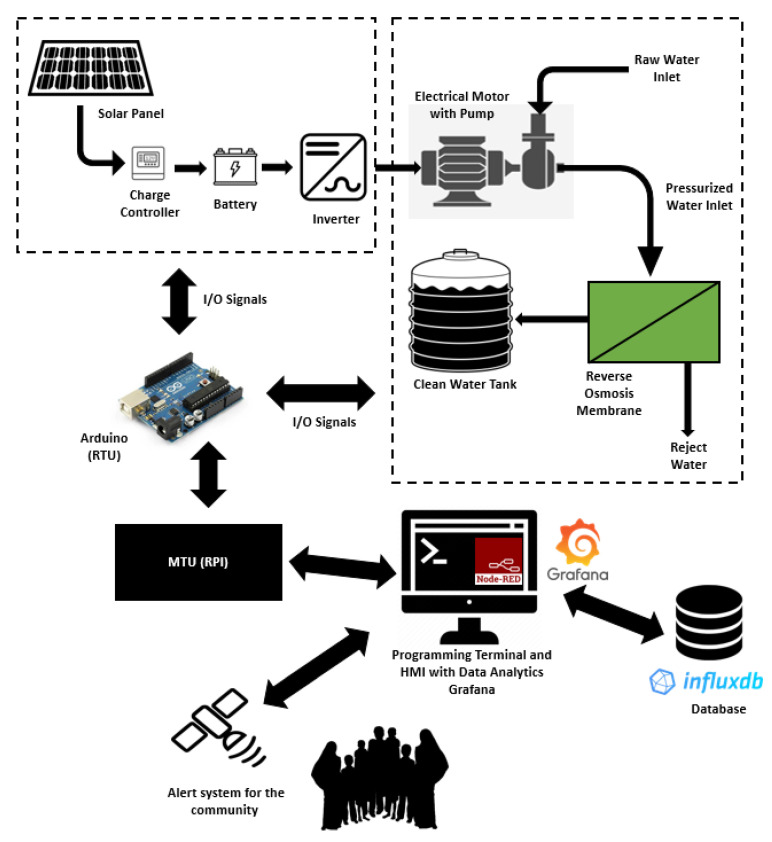
Proposed SCADA System for a Solar-powered Reverse Osmosis Desalination System Architecture.

The designed open-source-based SCADA system is depicted in Figure 1. The system can be elaborated in two parts which include an electrical power system and a water desalination system. In an electrical power system, solar panels are used to charge the battery through the charge controller, which ensures the maximum power point is tracked. Full details of system design and dynamic modeling may be found in our earlier published work [5,6]. This electrical power is then fed to the inverter to run the electric motor. For the reverse osmosis desalination system, raw water is taken and passed through the high-pressure pump to achieve desired water pressure to enter the reverse osmosis desalination membrane. The membrane is the most important component in the reverse osmosis system; water molecules can pass through its small pores, and contaminants are prevented from entering. When water passes through the membrane, the dirty water is collected on the more concentrated side, whereas the clean water is collected on the less concentrated side [30]. Afterward, the clean water is stored in a water tank in order to ensure an uninterrupted supply of water to the community. In order to measure all the important parameters from the complete system, several sensors are used. For demonstration purposes, Arduino Mega 2560 microcontroller has used that act as the RTU for all input and output communications. The sensor data taken from the electrical system includes solar panel voltage and current, whereas the inlet water pressure, inlet water temperature, pump pressure, outlet pressure, and clean water tank level are measured for the reverse osmosis desalination system. The pressure pump control is also provided to ensure in case of any fault that, the pump is turned off. This study used a low-cost computer running raspberry Pi Software x86 with a 32-bit Debian operating system and the 5.10 kernel installed on an x86-64-bit processor. The computer has an Intel i5, 4-core central processing unit (CPU) with 4 GB of RAM. Inside the programming terminal, Node-Red version 3.0.2 and node.js version 16.16.0 with dashboard version 3.1.7 is installed for programming and HMI design. For the purpose of the database, InfluxDB version 1.5.3 is installed on the computer for storing data locally. In order to have data visualization and analysis, Grafana version 7.4.5 is installed on the same programming terminal. The system was programmed to provide important alerts to the community using the GSM system. 

## 4. Components of the Designed System

Systematically, all field device data is collected by the RTU and sent to the Raspberry Pi-Node-Red-based Programming terminal. After that, the Node-Red-based graphical user interface utilizes the data for displaying several parameters, whereas control buttons and community notifications are also available. Further, the data is stored in the local Influx DB database and used by Grafana for historical data analytics. The comprehensive detail of each component used in the design is described below.

### 4.1. Field Instrument Devices

Field Instrument devices play an essential role in the operation of the system. The field instrument devices are capable of measuring what is occurring on the ground. The values are taken from the FIDs, fed to RTU, and eventually sent to the open-source programming terminal. This designed system is divided into three sections based on physical stimulus, measurements, and actuation, as stated below.

- Electrical System FIDs
- Reverse Osmosis Desalination System FIDs
- Actuators and Simulators FIDs

All the FIDs used in the system are summarized in Table 1 with their manufacturer, model, and desired function.

#### 4.1.1. Electrical System FIDs

**Figure 2:**
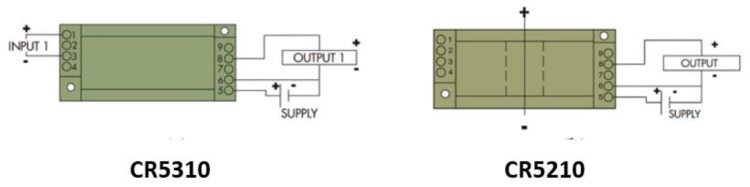
Current and voltage sensor wiring diagram [31,32].

CR5310 [31] and CR5210 [32] are used as the DC voltage and current sensors, respectively. The CR5310 sensor provides output in the range of 0–5 V DC and is directly proportional to the input range of 0–600 V DC whereas the CR5210 sensor has a current input range of 200 A DC. These sensors have a very important feature of isolation from the input side to the output side, which will make sure that the RTU is not damaged in case of an overvoltage or current situation. In order to operate both sensors, a working voltage of 24 V DC is required. Figure 2 shows the wiring diagram of the sensor. The technical specifications for CR5310 and CR5210 are also summarized in Table 2.

#### 4.1.2. Reverse Osmosis System FIDs

**Figure 3:**
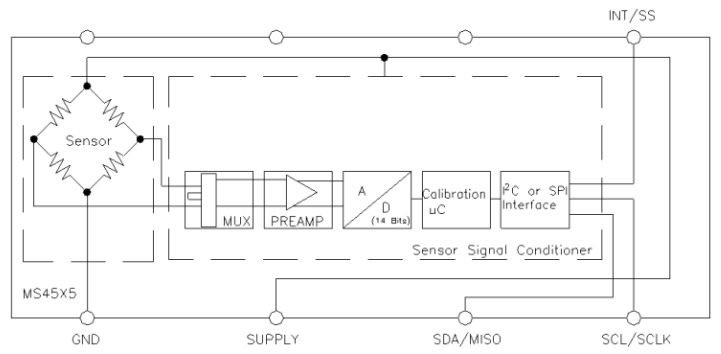
Pressure sensor internal block diagram [33].

**Figure 4:**
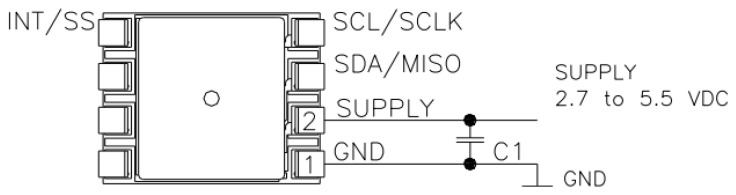
Pressure sensor connection diagram [33].

Inlet pressure, pump pressure, and outlet pressure are all measured by the same type of transducer. The TE Connectivity Measurements Specialties transducer [33] serves as the single solution for all pressure measurements. The transducer is a small PCB-mounted sensor. It is based on the modern CMOS sensor conditioning circuitry to create a low-cost, efficient sensor. The sensor is an 8-pin device with a pressure measurement port on top of it. Figure 3 illustrates the internal circuit of the sensor, whereas Figure 4 shows the connections.

Further technical descriptions of the sensor are summarized in Table 3.

**Figure 5:**
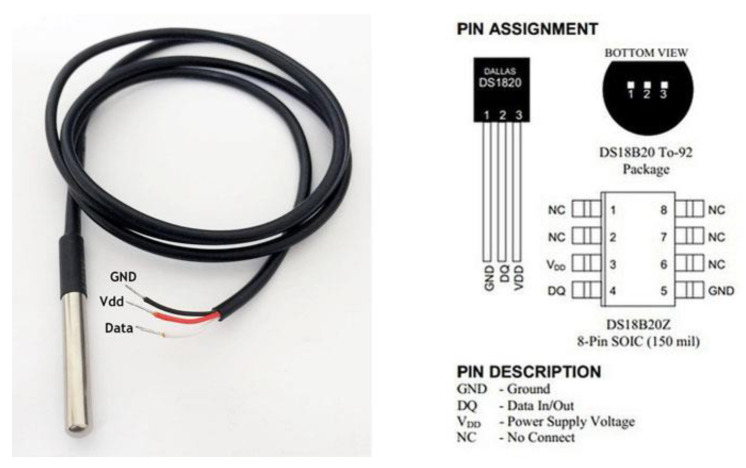
Temperature sensor DS18B20 physical and connection diagram [34].

The inlet temperature is measured using the Maxim DS18B20 [34] sensor, which is a one-wire 12-bit temperature sensor. The sensor can be powered by 3 to 5 V DC and can measure any temperature between −55 °C to 125 °C. The sensor has a waterproof packing making it super useful to use in wet conditions and in water applications. Figure 5 shows the physical and connection diagram for the sensor. 

A summary of the sensor’s technical specifications is provided in Table 4.

**Figure 6:**
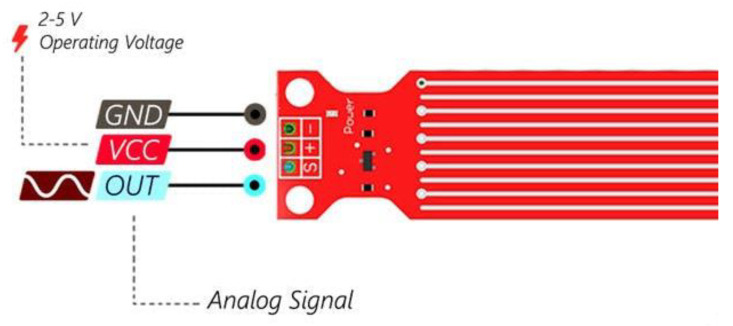
Water level sensor physical and connection diagram [35].

The clean water tank level is measured using the water sensor module by SongHe [35]. The sensor is easy to integrate, compact, lightweight, and has traces of copper to identify the level of water. The sensor works by interlacing grounded traces with the sensor traces by connecting them to the ground. Figure 6 represents the connection diagram for the sensor. 

A summary of the sensor’s technical specifications can be found in Table 5.

#### 4.1.3. Actuators and Simulators FIDs

**Figure 7:**
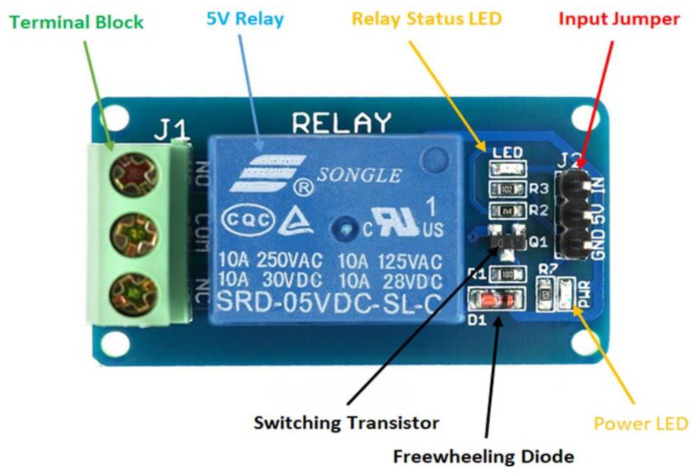
Relay module physical hardware and connection points [36].

The relay module (Lychee Limited 06-061-024) [36] is used for controlling the main pump from the RTU. The RTU provides 0 V DC for the off signal and 5 V DC for the on signal to the relay module, which in turn will turn the pump on or off. The device has the capability to be used in normally open or normally closed configurations. Figure 7 demonstrates all components installed on the device.

Further, Table 6 below describes the technical details of the relay.

**Figure 8:**
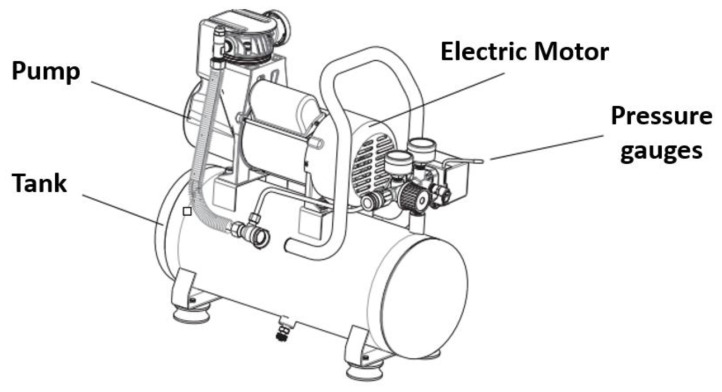
Major parts of the compressor [37].

The three master craft compressors [37] are used to simulate the pressure values of the system. These compressors feature an oil-free design which ensures less maintenance. It is a single-hand operation device with a quick setup. The rubber feet of the compressor allow more stable operation. The unit has local pressure gauges to check the pressure readings. Figure 8 reveals the major parts of the compressor device, which includes a pump for building up the pressure and discharging it into the tank, an electric motor for the rotation of the pump, a tank to store the compressed air, and a few pressure gauges to show the discharge and inlet pressures.

Table 7 summarizes additional technical specifications of the compressor.

### 4.2. Remote Terminal Unit (Arduino Mega 2560)

The Arduino Mega 2560 [38] microcontroller board is used as the RTU. It is based on the Atmega 2560 controller chip. Among the 54 digital input and output pins on the board, 14 are capable of acting as pulse width modulation (PWM) output pins. Aside from that, the board features 16 analog inputs and four universal asynchronous receiver transmitter pins (UARTs). The board consists of a 16 MHz crystal oscillator provided with a standard universal serial bus (USB) connection, a reset button, an in-circuit serial programming (ICSP) header, and a power jack. Further technical specifications of the Arduino Mega 2560 are summarized in Table 8.

The Arduino Mega is configured using the Arduino Integrated Development Environment (IDE). The IDE is a versatile editor where the programmer can install different libraries, make their own programs, and debug them to check for errors. The code written in IDE is called sketches. In addition to C++, Arduino codes contain additional methods and functions that enhance their functionality. The IDE has a serial monitor option where the programmer can interact with the board for real-time monitoring and debugging. The serial plotter is another important feature of the IDE, where real-time graphs of your serial data can be plotted, and waveforms can be analyzed. The library structure of any sketch is a folder comprised of files with C++ Code (.cpp) and header files (.h). After you have coded your desired task in the IDE, you can compile the code to check for errors and upload the code to the Arduino mega 2560 board using a standard USB serial connection and run it physically.

### 4.3. Main Terminal Unit

This system relies heavily on the main terminal unit. That is responsible for data acquisition, programming, data visualization, and data storage. In order to understand this unit, it can be divided into two categories which include hardware and software. An old computer is used as the physical hardware on which Raspbian as, the operating system, is running to control and monitor the system. The details of both categories are further explained below sub-sections.

#### 4.3.1. Hardware (Physical Device)

An old MacBook Air (13-inch, mid-2012) is used as the primary hardware for the main terminal Unit. The unit has 128 GB of flash storage and has 1.8 GHz dual-core Intel Core i5 processor. The unit has 4 GB of RAM, have an Intel HD Graphics 4000 Card with a 1400 × 900 screen ratio for quality display. Further, the unit has an SD card slot, 2 USB 3.0 ports, a thunderbolt, Magsafe 2.0, and a headphone port. The device complies with IEEE standard 802.11n and 802.11a/b/g protocols. The device has a compact 50-watt-hour lithium polymer battery which can run the system on standby for 30 days and up to 7 h when using wireless and the web. The device operates on 100–240 V AC with a frequency range of 50–60 Hz inside an operating temperature range of 10–35 °C. As a part of this study, we have configured Raspberry Pi OS (64-bit) on a MacBook Air.

#### 4.3.2. Software (Operating System, Applications, and Database)

For a better understanding of each component within the software package, the system can be divided into three parts which are stated below and explained further in subsections.

- Operating System
- Applications
- Database

##### Operating System

Raspberry is a small single-board computer that can be used widely with several modifications. It provides a control for several applications extending from small-scale systems to large industrial processes. In order for the system to run, it requires an operating system (OS), normally Raspbian, to execute the operations. The biggest advantage of MTU is that it is highly flexible, and any computer can be transformed into the raspberry pi operating system (RPI). The OS installed for the execution of further tasks is Debian GNU/Linux 11.0 (bullseye). The OS has a newer theme compared to the previous Linux OS. It further has upgraded the Linux kernel from 4.19 to 5.10, which means it can provide better hardware support along with performance improvement. Linux Kernel 5.10 is a long-term support kernel that will be supported until December 2026. The OS further allows driverless printing and scanning facilities with improved security as the default encryption algorithm has been replaced by Yescrypt. With all these features, easy installation, and low space, the OS was installed on the hardware. The authors of [39] also used raspberry pi to host a private server.

##### Applications

The application software used for the development of the system includes Node-Red and Grafana. The Node-Red editor uses flow-based visual programming, originally developed by IBM’s emerging technology services team. This software is now part of the OpenJS foundation. Node-Red is open-source, where you can develop codes and link the physical hardware by online coding. It contains a browser-based programming platform where you can install several libraries to connect and communicate with the physical world. The major advantage of using Node-Red is that it uses an easy-to-wire programming language where you can visualize the node flows and how the code sequence will execute. In programming, there are “black boxes” which you call “nodes” that have a well-defined purpose and can be linked by wire programming for data flow. The programming embedded with the visual representation of the flows allows the software to be used by a variety of users. The web-based programming technology is based on Node.js for editing the flows. New nodes can be easily imported as the software has a huge community where developers can program their nodes and share them with the rest of the community. The new flow can then be easily shared as javascript object notation (JSON) files. The Node-Red version 3.0.2 with the node.js version 16.16.0 was used for the development of the system. In [40], the authors used Node-Red as a preferred IoT platform and stressed the importance of configuring the system on a local machine to ensure system security and privacy.

One of the biggest features of the Node-Red is the dashboard module. This allows users to quickly create a live dashboard for monitoring parameters. 

The Node-Red dashboard version 3.1.7 was installed for the development of the system. The other most important software application used for the development of the system is Grafana. For every database, Grafana provides analytics and monitoring functionality. The Grafana version 7.4.5 was used for the creation of this system. With the help of this software, users can write queries, visualize, create alerts, and analyze data stored anywhere. It allows the user to create, explore and share all the data through attractive dashboards, which makes decisions easier and better. With these capabilities, user can understand all relevant data, create a relationship between them, and most importantly, helps in identifying the root cause analysis for incidents as quickly as possible. 

##### Database

A database is an organized collection of structured information that is saved in electronic form inside the storage of a computer system. The database is normally controlled by the by management software that manages the saved information inside the storage location. InfluxDB version 1.5.3 is used as the database for this system. It provides a smart data platform with everything to create a time series database. It allows users to create multi-tenanted time series databases, dashboard tools, data processing, and monitoring. The extensive community groups allow developers to collaborate and build efficient ways to store, manage and retrieve data.

## 5. Implementation Methodology

In implementing the design, all the FIDs need to be connected to the RTU. Table 9 below summarizes all the FIDs interconnection to the RTU pins. 

The FIDs data coming to the RTU is sent to the MTU using the serial communication between RTU and MTU. The RTU is programmed with the firmata protocol to make communication easier and faster. Firmata is a protocol for communicating with microcontrollers from software based on a computer. The protocol is loaded on the firmware of the microcontroller so that the microcontroller act as the support for that package. The most commonly implemented versions of firmata are for Arduino and Spark.io. After the communication between the RTU and MTU is achieved using the firmata protocol, Node-Red programming is carried out. The Node-Red execution and programming are explained using a series of algorithms. Algorithm 1 explains the field sensor data acquisition, display in the Node-Red dashboard, and storage in the InfluxDB database. The Algorithm check where the system and service are running correctly. Once that step is verified, the system reads the data from the RTU and displays it to the live dashboard at the same time, store it locally on the database. Algorithm 2 elaborates the System operation where the system again checks the status of services running and allows the user to press buttons from the dashboard for starting, stopping, and carrying out operational tasks. Algorithm 3 outlines the alert system to the community. Similar to the first two algorithms, the system checks the status of services running and gives alerts to the community based on the condition of the system. The important alerts sent information about the low and high water levels in clean tanks, started and stopped the system and pumps, and scheduled maintenance and restoration of the system etc. After the data acquisition is completed, the data is stored in the InfluxDB database and later called by Grafana for data analytics and visualization.
**Algorithm 1:** Field Sensor Data Acquisition, Storage, and Display   Initialization;     1. Start Service Node-Red     2. Check Service Node-Red     3. If Running          a. Open localhost:1880          b. While Read and store data from pins A0, A1, A2, A3, A4, A5 and A6 using /dev/ttyACM0               i. Send A0, A1, A3 and A6 to chart display               ii. Send A2, A4 and A5 to gauge display               iii. Send A0 to Solar Current tag database               iv. Send A1 to Solar Voltage tag database               v. Send A2 to Inlet Pressure tag database               vi. Send A3 to Inlet Temperature tag database               vii. Send A4 to Pump Pressure tag database               viii. Send A5 to Outlet Pressure tag database               ix. Send A6 to Tank level tag database          c. End     4. Else Check System     5. End

**Algorithm 2:** System Operation   Initialization;     1. Start Service Node-Red     2. Check Service Node-Red     3. If Running          a. Go to localhost:1880/ui          b. Click System On button          c. Click Pump On button     4. Else          a. Click Pump Off button     5. Else If          a. Click System Off button          b. Pump Off button Automatically activated by interlocking     6. End Check System     7. End

**Algorithm 3:** Alert System for Community   Initialization;     1. Start Service Node-Red     2. Check Service Node-Red     3. If Running          a. Open localhost:1880/ui          b. Set Clean Water Level Lower Set Point Variable          c. Set Clean Water Level Upper Set Point Variable          d. While Read Dashboard button status and Pin A6               i. If A6 value is less than the Clean Water Level Lower Set Point               1. Send Message to community: “Warning, The clean water level availability is very low”               ii. End               iii. If A6 value is greater than the Clean Water Level Upper Set Point                    1. Send Message to community: “Notification, The clean water level availability is very High”               iv. End               v. If System ON button is active                    1. Send Message to the community “System Status: The System has been turned ON!”               vi. Else If System OFF button is active                    1. Send Message to the community “System Status: The System has been turned OFF!”               vii. Else If Pump ON button is active                    1. Send Message to the community “System Status: The Main Pump is running!”               viii. Else if Pump OFF button is active                    1. Send Message to the community “System Status: The Main Pump turned OFF!”               ix. Else if Send Maintenance Alert Button is active                    1. Send Message to the community “Alert, The System will remain non-operational today because of maintenance activity. The system will be back online tomorrow. Apologies for the inconvenience caused!”                x. Else if Send System Normalization Alert Button is active                    1. Send Message to the community “Alert, The system has been restored and fully functional after the maintenance shutdown”               xi. End          e. End     4. End Check System     5. End

JavaScript code for Algorithms 1 and 3 is presented in Appendix A. 

## 6. Prototype Design and Results

**Figure 9:**
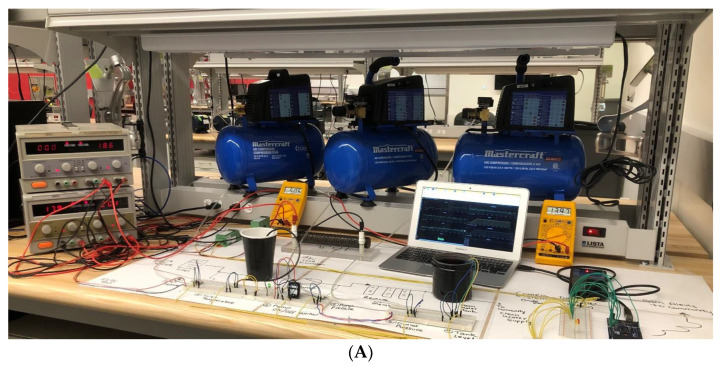

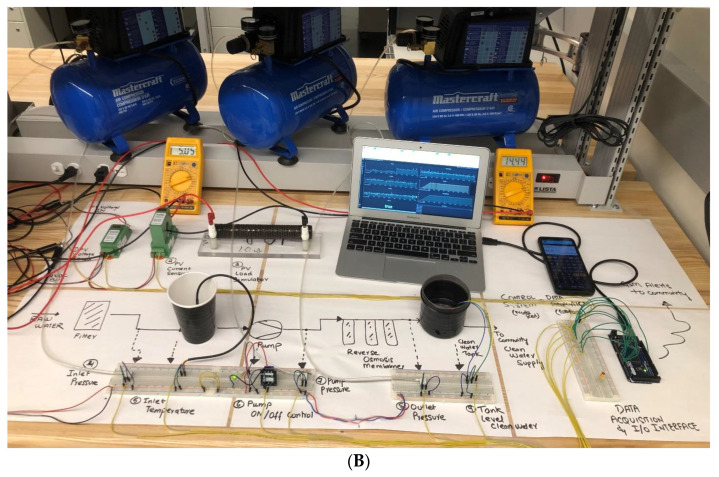
(**A**) Experimental setup of the system (Front View). (**B**) Experimental setup of the system (Top View).

The proposed experimental system setup is presented in Figure 9A (Front view) and Figure 9B (top view) using the aforementioned hardware and operating standards. As shown in the figure, all the field devices are wired to the RTU, which is then connected to the MTU (MacBook Pro) using the USB cable. A 24 V DC and 5 V DC bus was created to power all the field devices. Two ABRA DC power supplies, AB-3300 were used to generate the desired voltage, which has the functionality to provide variable voltage and current. The power supply has a voltage range from 0–60 V DC. A 10 Ohm, 300-Watt power rheostat is used as a load to generate the solar current. The variable power supply is further used to generate the required solar voltage. The three compressors are used to simulate the pressure values at different stages of the process. Two water cups filled with water are used to measure the water temperature and level in the hardware prototype. The Metex M3800 digital millimeters were connected to measure voltages and current on the point of interest to cross-check the measurement of field parameters.

**Figure 10:**
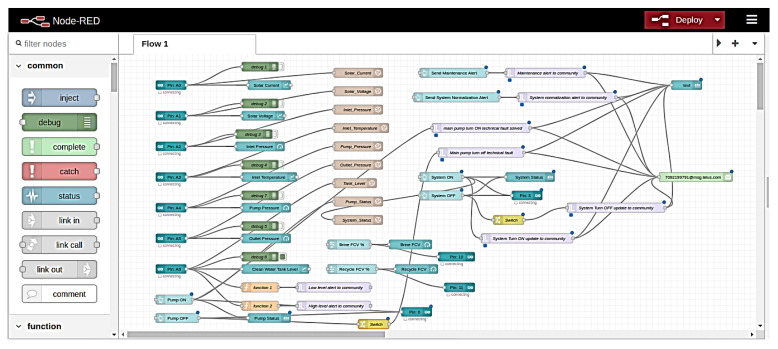
Node-Red programming of the system.

All system was powered up, and steps were performed to check the system’s operation. Firstly, Arduino IDE is opened in the MTU, and firmata is uploaded to the microcontroller. Node-Red Programming is carried out in the web-based programming environment, and all logical operations were constructed as flow sequences. Figure 10 shows the Node-Red programming flow diagram (code provided in Appendix A). Flow-based servers and UIs were developed using Node-Red visual programming language [41,42]. 

**Figure 11:**
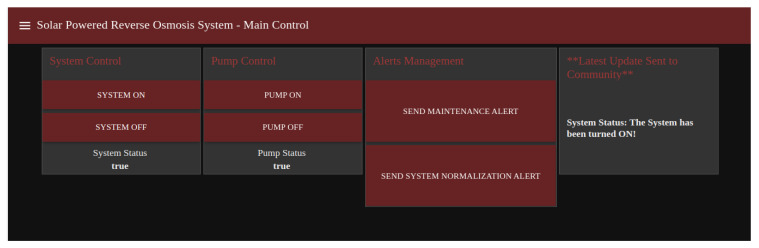
Node-Red Dashboard for operation.

The Node-Red dashboard was created with all major functions required for smooth system operation and sending messages and alerts to the community. Figure 11 shows the Node-Red Operational dashboard available for the user.

**Figure 12:**
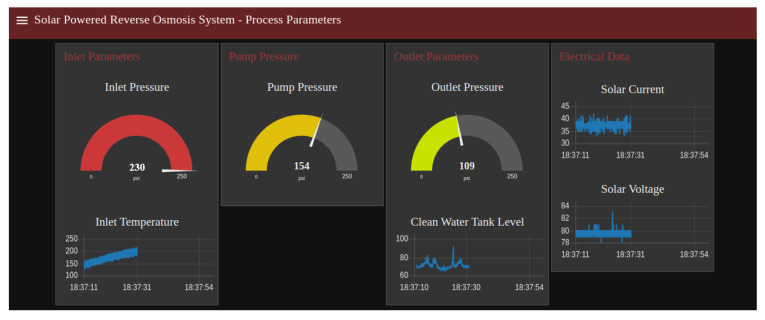
Node-Red Dashboard for real-time monitoring.

The Node-Red real-time monitoring dashboard was created to show the live values of several process parameters. Scaling of the analog return values was performed to read the actual pressure. Figure 12 shows the Node-Red real-time monitoring dashboard.

**Figure 13:**
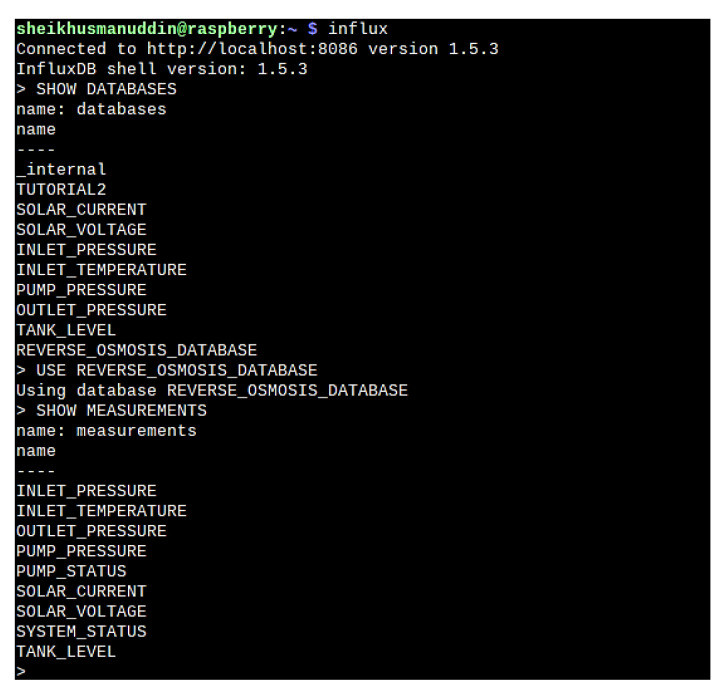
InfluxDB database tag generation.

In order to store the data on a local server, InfluxDb is used as the database, and tags of all process parameters were created. Figure 13 shows the database structure and tags generated in the system.

**Figure 14:**
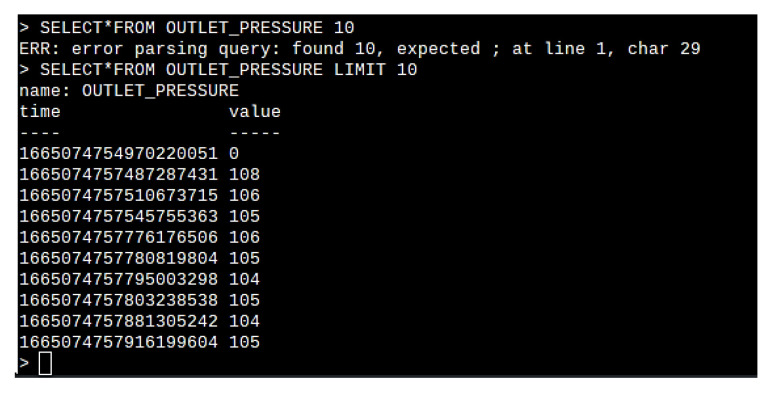
Verification of database functionality.

Figure 14 further illustrates the last values stored inside the database that is directly coming from the Node-Red programming to verify the operational functionality of the database.

**Figure 15:**
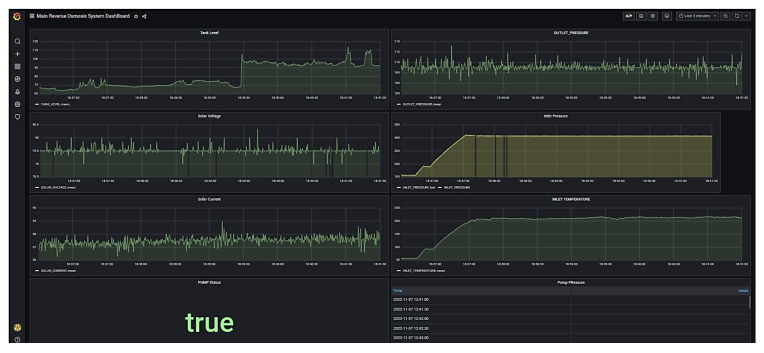
Grafana-based dashboard for historical data trending and data analytics.

Finally, the stored database is called inside the Grafana web-based data analytics tool. Figure 15 demonstrates the dashboard generated in the Grafana visualization tool for viewing historical data and carrying out data analysis. 

**Figure 16:**
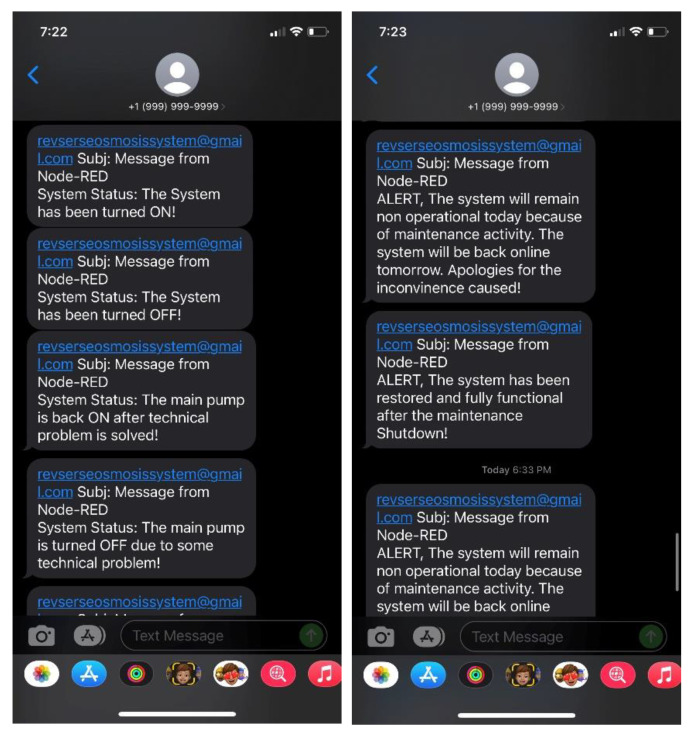
System status and alert messages received on a mobile phone.

Alert system functionality was also verified by using the Node-Red online, operational dashboard, and messages were received on the mobile phone from Node-Red. Figure 16 shows all the messages received.

## 7. Discussion

This section aims to highlight some of the main features and benefits of the open-source-based SCADA system of solar-powered reverse osmosis system for a community realized following successful testing.

- System Configuration: The System is designed in a unique configuration where a web-based approach is used for both logical programming and data analytics.
- System safety: As a measure of system safety, the field devices are isolated using an RTU to ensure that the system side is protected in the event of abnormal conditions.
- Open Source: The complete system is based on free and open-source software that can be easily installed on any operating system. There is no license fee or yearly fee associated with the operation of the system. Hence, eliminating the operational cost of the system.
- Availability and Reliability of System: Since all the components used in the system are easily available in the market and all work is done locally, including the creation of a local database ensures the user has a continuously reliable and available system.
- Data Acquisition, Monitoring, and Control: All data is locally collected using the RTU and monitored on a web-based monitoring and control system for operation and maintenance.
- Data Storage: Data is locally stored in a local server using InfluxDB, where using the Grafana-based system you can view historical data.
- Alert System for the Community: All important alerts regarding the system are promptly communicated to community members that rely on this water source for their daily needs. Consequently, if there is an abnormal water situation, the community is informed and can respond appropriately.
- User-Friendly Dashboard: An easy-to-use dashboard provides easy access to real-time and historical data.
- Security: Due to the fact that the system stores data locally and does not transmit any information to a remote server, the system is a fully private and secure system.
- Easy-to-Use System: The system will require one-time training for any operator and can be easily used by any user.
- Data Analytics: The System has the capability to carry out extensive data analytics as the data is now available on at Grafana server that has very high processing and data analytics power.
- Comprehensive guide for future research: The results of this research will serve as a guide for the development of clean water systems that are easy to install in any community, regardless of whether it is connected to the grid or not.
- System Limitations: The SCADA system is designed for the selected RO system. It has some limits, e.g., selected sensors could be used for the selected RO system; after a power interruption SCADA server needs to be restarted by switching on the computer and running node-Red and Grafana manually. Another system limit is that it lacks RO system user feedback. The system will send an alert, but it cannot determine whether users received that alert or not. Users cannot send a message/email to check the system status. More features may be added in future versions of this SCADA system.

## 8. Conclusions

1. Globally, the use of clean energy and clean water scarcity remain the two biggest challenges. This paper discusses an elaborate system design that ensures the reverse osmosis desalination system is powered exclusively by renewable energy. There is a detailed description of the majority of field instrument devices required to monitor and control the system in the paper. Field devices are integrated with RTUs to acquire data, which is then transmitted to the MTUs for processing and control. A highly interactive and real-time monitoring and control HMI was also developed with the use of the Node-Red visual programming language. Furthermore, the most challenging aspect of the operation of any system is to ensure that its security is not compromised. Thus, in order to ensure the security of the system, InfluxDB is used as the database and is executed locally on the same MTU, storing the data on the local server so that only authorized personnel will be able to access it. As data-driven decisions and predictive analyses play a pivotal role in increasing the efficiency and performance of any system, Grafana is subsequently deployed on the same MTU as a web-based data analytics and predictive analytics application. A major advantage of all these applications is that they are free of charge and do not require a license or an annual fee, as well as being open source with easy access to updates. As a final point, the most important gap in all such systems and previous research work was the absence of a user alert system, as in a typical situation, users would have to visit the system in person to know the status and quantity of water available.
2. Research such as this can be applied to many existing reverse osmosis-based desalination plants in many parts of the world to ensure that data analytics can be used to make better data-driven decisions. It offers a programming-based integration method without any external hardware for extensive alert systems that can be used separately with a variety of systems. Renewable energy is used throughout the system, thereby contributing to its environmental friendliness. Based on the testing of the system, it has been demonstrated that it can be used to remotely control and log data accurately in real time.

## Figures and Tables

**Table 1:** Sensor manufacturer, models, and function.

Manufacturer	Model	Function	References
CR Magnetics	CR5310	DC Voltage Transducer	[31]
CR Magnetics	CR5210	DC Current Transducer	[32]
TE Connectivity Measurements Specialties	4525-15AP	Inlet Pressure Transducer	[33]
Maxim	DS18B20	Inlet Temperature Transducer	[34]
TE Connectivity Measurements Specialties	4525-15AP	Pump Pressure Transducer	[33]
TE Connectivity Measurements Specialties	4525-15AP	Outlet Pressure Transducer	[33]
SongHe	B07THDH7Y4	Clean Water Tank Level Transducer	[35]
Lychee Limited	06-061-024	Relay Module for Pump On/Off	[36]
Mastercraft	2 Gallon Air	Compressor for Inlet Pressure Simulation	[37]
Mastercraft	2 Gallon Air	Compressor for Pump Pressure Simulation	[37]
Mastercraft	2 Gallon Air	Compressor for Outlet Pressure Simulation	[37]

**Table 2:** Technical Specification of current and voltage sensor [31,32].

Specification	Value	Units
Accuracy	1.0	%
Linearity	10 to 100	% FS
Thermal Drift	500	PPM/C
Operating Temperature	0 to 50	C
Response Time	250	Ms
Supply Voltage	24	V DC
Supply Current	35	mA

**Table 3:** Technical Specification of pressure sensor [33].

Specification	Value	Units
Supply Voltage	2.7 to 5.5	V DC
Output Current	3	mA
Over Pressure	300	Psi
Accuracy	−0.25 to 0.25	% of Span
Operating Temperature	−25 to 105	C
Response Time	0.5	ms

**Table 4:** Technical Specification of temperature sensor [34].

Specification	Value	Units
Supply Voltage	3.0 to 5.5	V DC
Thermometer Error	+/−2	C
Standby Current	750	nA
Active Current	1	mA

**Table 5:** Technical Specification of water level sensor [35].

Specification	Value	Units
Operating Voltage	3.0 to 5.5	V DC
Working Current	<20	mA
Working Temperature	10 to 30	C
Output Voltage	0 to 4.2	V DC

**Table 6:** Relay module technical specifications [36].

Specification	Value	Units
Maximum Output	30	V DC
	250	V AC
Voltage Input	5	V DC
Operation Time	10	ms
Release Time	5	ms
Maximum On–Off Switching	30	Operations/min
Operating Temperature	−25 to 70	C

**Table 7:** Technical specifications of compressor [37].

Specification	Value	Units
Power	1.0	Hp
Voltage Input	120	V AC
Frequency	60	Hz
Duty Cycle	50	%
Tank Size	3	U.S. Gallons
Pressure Range	0–125	PSI
Compressor Capacity	3 @ 40 PSI 2 @ 90 PSI	CFM

**Table 8:** Technical specifications of RTU [38].

Specification	Value	Units
Operating Voltage	5	V DC
Input Voltage (Recommended)	7–12	V DC
Input Voltage (Limits)	6–20	V DC
Digital I/O	54	Pins
Analog Input	16	Pins
Current Per I/O Pin	40	mA
Flash Memory	256	KB
Static Random Access Memory	8	KB
Electrically Erasable Programmable Read-Only Memory	4	KB
Clock Speed	16	MHz

**Table 9:** Field instrumentation devices interconnection.

Sr. #	Specification	Value	Arduino Pin
1	Solar Voltage Senor	Analogue	A0
2	Solar Current Sensor	Analogue	A1
3	Inlet Pressure	Analogue	A2
4	Inlet Temperature	Analogue	A3
5	Pump Pressure	Analogue	A4
6	Outlet Pressure	Analogue	A5
7	Clean Water Tank Level	Analogue	A6
8	Pump On/Off Control Relay	Digital	D6

## Data Availability

All the data is provided already in references and Appendix A.

## Abbreviations

The following major abbreviations are used in this manuscript.

SCADA	Supervisory Control and Data Acquisition
HMI	Human Machine Interface
PLC	Programmable Logic Controller
MTU	Master Terminal Unit
RTU	Remote Terminal Unit
FID	Field Instrument Device
PV	Photovoltaic
GSM	Global System for Mobile Communication
RO	Reverse Osmosis
DCS	Distributed Control System

## Appendix A

[{“id”:“e19cb62755280239”,“type”:“ui_gauge”,“z”:“66d22c941c2b4f7a”,“name”:“”,“group”:“46fd27459ff5a257”,“order”:1,“width”:0,“height”:0,“gtype”:“gage”,“title”:“Inlet Pressure”,“label”:“psi”,“format”:“{{value}}”,“min”:0,“max”:“250”,“colors”:[“#00b500”,“#e6e600”,“#ca3838”],“seg1”:“”,“seg2”:“”,“className”:“”,“x”:460,“y”:300,“wires”:[]},{“id”:“daca1cc85c6fe3f6”,“type”:“ui_chart”,“z”:“66d22c941c2b4f7a”,“name”:“”,“group”:“67e5b6569270f44d”,“order”:1,“width”:0,“height”:0,“label”:“Solar Current”,“chartType”:“line”,“legend”:“false”,“xformat”:“HH:mm:ss”,“interpolate”:“linear”,“nodata”:“”,“dot”:false,“ymin”:“”,“ymax”:“”,“removeOlder”:“20”,“removeOlderPoints”:“”,“removeOlderUnit”:“1”,“cutout”:0,“useOneColor”:false,“useUTC”:false,“colors”:[“#1f77b4”,“#aec7e8”,“#ff7f0e”,“#2ca02c”,“#98df8a”,“#d62728”,“#ff9896”,“#9467bd”,“#c5b0d5”],“outputs”:1,“useDifferentColor”:false,“className”:“”,“x”:490,“y”:100,“wires”:[[]]},{“id”:“e437c172a99eb165”,“type”:“ui_chart”,“z”:“66d22c941c2b4f7a”,“name”:“”,“group”:“67e5b6569270f44d”,“order”:2,“width”:0,“height”:0,“label”:“Solar Voltage”,“chartType”:“line”,“legend”:“false”,“xformat”:“HH:mm:ss”,“interpolate”:“linear”,“nodata”:“”,“dot”:false,“ymin”:“”,“ymax”:“”,“removeOlder”:“20”,“removeOlderPoints”:“”,“removeOlderUnit”:“1”,“cutout”:0,“useOneColor”:false,“useUTC”:false,“colors”:[“#1f77b4”,“#aec7e8”,“#ff7f0e”,“#2ca02c”,“#98df8a”,“#d62728”,“#ff9896”,“#9467bd”,“#c5b0d5”],“outputs”:1,“useDifferentColor”:false,“className”:“”,“x”:470,“y”:200,“wires”:[[]]},{“id”:“2fbf9910950159a8”,“type”:“ui_button”,“z”:“66d22c941c2b4f7a”,“name”:“”,“group”:“1aa03ee7932a5729”,“order”:1,“width”:0,“height”:0,“passthru”:false,“label”:“System ON”,“tooltip”:“”,“color”:“”,“bgcolor”:”“,”className”:”“,”icon”:“”,“payload”:“true”,“payloadType”:“bool”,“topic”:“topic”,“topicType”:“msg”,“x”:770,“y”:620,“wires”:[[“87625954e815df82”,“4f720392fecedbda”,“f4c496d296a96d68”,“9c48f7d63d15a4bb”]]},{“id”:“87625954e815df82”,“type”:“ui_text”,“z”:“66d22c941c2b4f7a”,“group”:“1aa03ee7932a5729”,“order”:3,“width”:0,“height”:0,”name“:”“,“label”:“System Status”,“format”:“{{msg.payload}}”,“layout”:“col-center”,”className”:”“,”x”:1160,”y”:420,”wires”:[]},{“id”:”6bc9509d24a0a20b”,”type”:”ui_button”,”z”:”66d22c941c2b4f7a”,”name”:”“,”group”:”1aa03ee7932a5729”,”order”:2,”width”:0,”height”:0,”passthru”:false,”label”:”System OFF”,”tooltip”:”“,”color”:”“,”bgcolor”:”“,”className”:”“,”icon”:”“,”payload”:”false”,”payloadType”:”bool”,”topic”:”topic”,”topicType”:”msg”,”x”:790,”y”:680,”wires”:[[“87625954e815df82”,”4f720392fecedbda”,”ef2c1e7cda63d85f”]]},{“id”:”defe46ccc2366c96”,”type”:”arduino in”,”z”:”66d22c941c2b4f7a”,”name”:”“,”pin”:”2”,”state”:”ANALOG”,”arduino”:”6c6c1fbf4cf20203”,”x”:170,”y”:300,”wires”:[[“e19cb62755280239”,”02220de324010529”,”8721f1d8768e39d7”]]},{“id”:”19c09715074c7775”,”type”:”arduino in”,”z”:”66d22c941c2b4f7a”,”name”:”“,”pin”:”0”,”state”:”ANALOG”,”arduino”:”6c6c1fbf4cf20203”,”x”:170,”y”:100,”wires”:[[“daca1cc85c6fe3f6”,”d3a15fbc60c04614”,”d5995736ed3b77f9”]]},{“id”:”46abf98cd90fda1f”,”type”:”arduino in”,”z”:”66d22c941c2b4f7a”,”name”:”“,”pin”:”1”,”state”:”ANALOG”,”arduino”:”6c6c1fbf4cf20203”,”x”:170,”y”:200,”wires”:[[“e437c172a99eb165”,”92202aeeb389bd82”,”bd5c2393b7f0e08a”]]},{“id”:”4f720392fecedbda”,”type”:”arduino out”,”z”:”66d22c941c2b4f7a”,”name”:”“,”pin”:”3”,”state”:”OUTPUT”,”arduino”:”6c6c1fbf4cf20203”,”x”:1150,”y”:480,”wires”:[]},{“id”:”d3a15fbc60c04614”,”type”:”debug”,”z”:”66d22c941c2b4f7a”,”name”:”debug 1”,”active”:false,”tosidebar”:true,”console”:false,”tostatus”:false,”complete”:”payload”,”targetType”:”msg”,”statusVal”:”“,”statusType”:”auto”,”x”:460,”y”:40,”wires”:[]},{“id”:”92202aeeb389bd82”,”type”:”debug”,”z”:”66d22c941c2b4f7a”,”name”:”debug 2”,”active”:false,”tosidebar”:true,”console”:false,”tostatus”:false,”complete”:”payload”,”targetType”:”msg”,”statusVal”:”“,”statusType”:”auto”,”x”:460,”y”:160,”wires”:[]},{“id”:”02220de324010529”,”type”:”debug”,”z”:”66d22c941c2b4f7a”,”name”:”debug 3”,”active”:false,”tosidebar”:true,”console”:false,”tostatus”:false,”complete”:”payload”,”targetType”:”msg”,”statusVal”:”“,”statusType”:”auto”,”x”:440,”y”:260,”wires”:[]},{“id”:”5e8810bdc7f7f952”,”type”:”ui_chart”,”z”:”66d22c941c2b4f7a”,”name”:”“,”group”:”46fd27459ff5a257”,”order”:2,”width”:0,”height”:0,”label”:”Inlet Temperature”,”chartType”:”line”,”legend”:”false”,”xformat”:”HH:mm:ss”,”interpolate”:”linear”,”nodata”:”“,”dot”:false,”ymin”:”“,”ymax”:”“,”removeOlder”:”20”,”removeOlderPoints”:”“,”removeOlderUnit”:”1”,”cutout”:0,”useOneColor”:false,”useUTC”:false,”colors”:[“#1f77b4”,”#aec7e8”,”#ff7f0e”,”#2ca02c”,”#98df8a”,”#d62728”,”#ff9896”,”#9467bd”,”#c5b0d5”],”outputs”:1,”useDifferentColor”:false,”className”:”“,”x”:490,”y”:400,”wires”:[[]]},{“id”:”e2d85b9158150bb0”,”type”:”arduino in”,”z”:”66d22c941c2b4f7a”,”name”:”“,”pin”:”3”,”state”:”ANALOG”,”arduino”:”6c6c1fbf4cf20203”,”x”:170,”y”:400,”wires”:[[“5e8810bdc7f7f952”,”73dcc2130c33ef2d”,”7c7308e1a2d2e42b”]]},{“id”:”73dcc2130c33ef2d”,”type”:”debug”,”z”:”66d22c941c2b4f7a”,”name”:”debug 4”,”active”:false,”tosidebar”:true,”console”:false,”tostatus”:false,”complete”:”payload”,”targetType”:”msg”,”statusVal”:”“,”statusType”:”auto”,”x”:460,”y”:360,”wires”:[]},{“id”:”f74803955a449a63”,”type”:”arduino in”,”z”:”66d22c941c2b4f7a”,”name”:”“,”pin”:”5”,”state”:”ANALOG”,”arduino”:”6c6c1fbf4cf20203”,”x”:170,”y”:600,”wires”:[[“b82e51e77e312289”,”d7ce26664b57a248”,”5f631e9dfb48c4b3”]]},{“id”:”b82e51e77e312289”,”type”:”debug”,”z”:”66d22c941c2b4f7a”,”name”:”debug 5”,”active”:false,”tosidebar”:true,”console”:false,”tostatus”:false,”complete”:”payload”,”targetType”:”msg”,”statusVal”:”“,”statusType”:”auto”,”x”:460,”y”:560,”wires”:[]},{“id”:”d7ce26664b57a248”,”type”:”ui_gauge”,”z”:”66d22c941c2b4f7a”,”name”:”“,”group”:”7b6e6479fec5ed09”,”order”:1,”width”:0,”height”:0,”gtype”:”gage”,”title”:”Outlet Pressure”,”label”:”psi”,”format”:”{{value}}”,”min”:0,”max”:”250”,”colors”:[“#00b500”,”#e6e600”,”#ca3838”],”seg1”:”“,”seg2”:”“,”className”:”“,”x”:480,”y”:600,”wires”:[]},{“id”:”d0b1df3291c9fc22”,”type”:”ui_chart”,”z”:”66d22c941c2b4f7a”,”name”:”“,”group”:”7b6e6479fec5ed09”,”order”:2,”width”:0,”height”:0,”label”:”Clean Water Tank Level”,”chartType”:”line”,”legend”:”false”,”xformat”:”HH:mm:ss”,”interpolate”:”linear”,”nodata”:”“,”dot”:false,”ymin”:”“,”ymax”:”“,”removeOlder”:”20”,”removeOlderPoints”:”“,”removeOlderUnit”:”1”,”cutout”:0,”useOneColor”:false,”useUTC”:false,”colors”:[“#1f77b4”,”#aec7e8”,”#ff7f0e”,”#2ca02c”,”#98df8a”,”#d62728”,”#ff9896”,”#9467bd”,”#c5b0d5”],”outputs”:1,”useDifferentColor”:false,”className”:”“,”x”:510,”y”:700,”wires”:[[]]},{“id”:”1d1eb6ea05cda3a6”,”type”:”arduino in”,”z”:”66d22c941c2b4f7a”,”name”:”“,”pin”:”6”,”state”:”ANALOG”,”arduino”:”6c6c1fbf4cf20203”,”x”:170,”y”:700,”wires”:[[“d0b1df3291c9fc22”,”16ea9c239e8541da”,”4dc9a751abedb4f7”,”d4602af4e1d874d4”,”7d9422173149e539”]]},{“id”:”16ea9c239e8541da”,”type”:”debug”,”z”:”66d22c941c2b4f7a”,”name”:”debug 6”,”active”:true,”tosidebar”:true,”console”:false,”tostatus”:false,”complete”:”payload”,”targetType”:”msg”,”statusVal”:”“,”statusType”:”auto”,”x”:460,”y”:660,”wires”:[]},{“id”:”d3abc85e1c4d7c8d”,”type”:”arduino in”,”z”:”66d22c941c2b4f7a”,”name”:”“,”pin”:”4”,”state”:”ANALOG”,”arduino”:”6c6c1fbf4cf20203”,”x”:170,”y”:500,”wires”:[[“8d874a5410965b7e”,”a41e6eae6a20a2c9”,”5a9cc90c0e745f91”]]},{“id”:”8d874a5410965b7e”,”type”:”debug”,”z”:”66d22c941c2b4f7a”,”name”:”debug 7”,”active”:false,”tosidebar”:true,”console”:false,”tostatus”:false,”complete”:”payload”,”targetType”:”msg”,”statusVal”:”“,”statusType”:”auto”,”x”:460,”y”:460,”wires”:[]},{“id”:”a41e6eae6a20a2c9”,”type”:”ui_gauge”,”z”:”66d22c941c2b4f7a”,”name”:”“,”group”:”28bf606ea75adadd”,”order”:1,”width”:0,”height”:0,”gtype”:”gage”,”title”:”Pump Pressure”,”label”:”psi”,”format”:”{{value}}”,”min”:0,”max”:”250”,”colors”:[“#00b500”,”#e6e600”,”#ca3838”],”seg1”:”“,”seg2”:”“,”className”:”“,”x”:480,”y”:500,”wires”:[]},{“id”:”860a0379d1f61c58”,”type”:”ui_text”,”z”:”66d22c941c2b4f7a”,”group”:”84222b1d01e60129”,”order”:3,”width”:0,”height”:0,”name”:”“,”label”:”Pump Status”,”format”:”{{msg.payload}}”,”layout”:”col-center”,”className”:”“,”x”:490,”y”:860,”wires”:[]},{“id”:”7fe406631e7bccf1”,”type”:”arduino out”,”z”:”66d22c941c2b4f7a”,”name”:”“,”pin”:”6”,”state”:”OUTPUT”,”arduino”:”6c6c1fbf4cf20203”,”x”:970,”y”:840,”wires”:[]},{“id”:”2083b3147d799b10”,”type”:”ui_button”,”z”:”66d22c941c2b4f7a”,”name”:”“,”group”:”84222b1d01e60129”,”order”:1,”width”:0,”height”:0,”passthru”:false,”label”:”Pump ON”,”tooltip”:”“,”color”:”“,”bgcolor”:”“,”className”:”“,”icon”:”“,”payload”:”true”,”payloadType”:”bool”,”topic”:”topic”,”topicType”:”msg”,”x”:180,”y”:800,”wires”:[[“7fe406631e7bccf1”,”860a0379d1f61c58”,”dbe36386e2473459”,”56124fdcb3c89af7”]]},{“id”:”6fd982aa8ee1d761”,”type”:”ui_button”,”z”:”66d22c941c2b4f7a”,”name”:”“,”group”:”84222b1d01e60129”,”order”:2,”width”:0,”height”:0,”passthru”:false,”label”:”Pump OFF”,”tooltip”:”“,”color”:”“,”bgcolor”:”“,”className”:”“,”icon”:”“,”payload”:”false”,”payloadType”:”bool”,”topic”:”topic”,”topicType”:”msg”,”x”:190,”y”:860,”wires”:[[“7fe406631e7bccf1”,”860a0379d1f61c58”,”ae3af01f2e75c7c1”]]},{“id”:”1440feb1df6aa763”,”type”:”trigger”,”z”:”66d22c941c2b4f7a”,”name”:”Maintenance alert to community”,”op1”:”ALERT, The system will remain non operational today because of maintenance activity. The system will be back online tomorrow. Apologies for the inconvinence caused!”,”op2”:”0”,”op1type”:”str”,”op2type”:”str”,”duration”:”-12”,”extend”:false,”overrideDelay”:false,”units”:”hr”,”reset”:”false”,”bytopic”:”all”,”topic”:”topic”,”outputs”:1,”x”:1390,”y”:60,”wires”:[[“9bc5cf63289676e9”,”a23b424ce31ab227”]]},{“id”:”9bc5cf63289676e9”,”type”:”e-mail”,”z”:”66d22c941c2b4f7a”,”server”:”smtp.gmail.com“,”port”:”465”,”secure”:true,”tls”:true,”name”:”7092199791@msg.telus.com“,”dname”:”“,”x”:1880,”y”:400,”wires”:[]},{“id”:”34da0b71be09633e”,”type”:”ui_button”,”z”:”66d22c941c2b4f7a”,”name”:”“,”group”:”3d3554addfd3284e”,”order”:1,”width”:6,”height”:2,”passthru”:false,”label”:”Send Maintenance Alert”,”tooltip”:”“,”color”:”“,”bgcolor”:”“,”className”:”“,”icon”:”“,”payload”:”true”,”payloadType”:”bool”,”topic”:”topic”,”topicType”:”msg”,”x”:1090,”y”:60,”wires”:[[“1440feb1df6aa763”]]},{“id”:”a23b424ce31ab227”,”type”:”ui_text”,”z”:”66d22c941c2b4f7a”,”group”:”eabf10c445de3f5a”,”order”:1,”width”:5,”height”:3,”name”:”“,”label”:”“,”format”:”{{msg.payload}}”,”layout”:”col-center”,”className”:”“,”x”:1850,”y”:100,”wires”:[]},{“id”:”1fe08ceefa68bf24”,”type”:”trigger”,”z”:”66d22c941c2b4f7a”,”name”:”System normalization alert to community”,”op1”:”ALERT, The system has been restored and fully functional after the maintenance Shutdown!”,”op2”:”0”,”op1type”:”str”,”op2type”:”str”,”duration”:”-12”,”extend”:false,”overrideDelay”:false,”units”:”hr”,”reset”:”false”,”bytopic”:”all”,”topic”:”topic”,”outputs”:1,”x”:1460,”y”:140,”wires”:[[“a23b424ce31ab227”,”9bc5cf63289676e9”]]},{“id”:”bf31a2bffe60cd3e”,”type”:”ui_button”,”z”:”66d22c941c2b4f7a”,”name”:”“,”group”:”3d3554addfd3284e”,”order”:2,”width”:6,”height”:2,”passthru”:false,”label”:”Send System Normalization Alert”,”tooltip”:”“,”color”:”“,”bgcolor”:”“,”className”:”“,”icon”:”“,”payload”:”true”,”payloadType”:”bool”,”topic”:”topic”,”topicType”:”msg”,”x”:1100,”y”:140,”wires”:[[“1fe08ceefa68bf24”]]},{“id”:”4dc9a751abedb4f7”,”type”:”function”,”z”:”66d22c941c2b4f7a”,”name”:”function 1”,”func”:”if(msg.payload <= ‘37’)\n{\n msg.payload = ‘true’\n }\nelse\n{\n msg.payload = ‘false’\n}\nreturn msg;”,”outputs”:1,”noerr”:0,”initialize”:”“,”finalize”:”“,”libs”:[],”x”:460,”y”:760,”wires”:[[“0496fd60f52b4a6c”]]},{“id”:”0496fd60f52b4a6c”,”type”:”trigger”,”z”:”66d22c941c2b4f7a”,”name”:”Low level alert to community”,”op1”:”ALERT, The water level in the system is LOW!”,”op2”:”0”,”op1type”:”str”,”op2type”:”str”,”duration”:”-12”,”extend”:false,”overrideDelay”:false,”units”:”hr”,”reset”:”false”,”bytopic”:”all”,”topic”:”topic”,”outputs”:1,”x”:700,”y”:760,”wires”:[[]]},{“id”:”d4602af4e1d874d4”,”type”:”function”,”z”:”66d22c941c2b4f7a”,”name”:”function 2”,”func”:”if(msg.payload >= ‘55’)\n{\n msg.payload = ‘true’\n }\nelse\n{\n msg.payload = ‘false’\n}\nreturn msg;”,”outputs”:1,”noerr”:0,”initialize”:”“,”finalize”:”“,”libs”:[],”x”:460,”y”:820,”wires”:[[“0130930e7062d2d8”]]},{“id”:”0130930e7062d2d8”,”type”:”trigger”,”z”:”66d22c941c2b4f7a”,”name”:”High level alert to community”,”op1”:”ALERT, The water level in the system is HIGH!”,”op2”:”0”,”op1type”:”str”,”op2type”:”str”,”duration”:”-12”,”extend”:false,”overrideDelay”:false,”units”:”hr”,”reset”:”false”,”bytopic”:”all”,”topic”:”topic”,”outputs”:1,”x”:700,”y”:820,”wires”:[[]]},{“id”:”f4c496d296a96d68”,”type”:”trigger”,”z”:”66d22c941c2b4f7a”,”name”:”System Turn ON update to community”,”op1”:”System Status: The System has been turned ON!”,”op2”:”0”,”op1type”:”str”,”op2type”:”str”,”duration”:”-12”,”extend”:false,”overrideDelay”:false,”units”:”hr”,”reset”:”false”,”bytopic”:”all”,”topic”:”topic”,”outputs”:1,”x”:1330,”y”:600,”wires”:[[“9bc5cf63289676e9”,”a23b424ce31ab227”]]},{“id”:”efcaa6991f25e221”,”type”:”trigger”,”z”:”66d22c941c2b4f7a”,”name”:”System Turn OFF update to community”,”op1”:”System Status: The System has been turned OFF!”,”op2”:”0”,”op1type”:”str”,”op2type”:”str”,”duration”:”-12”,”extend”:false,”overrideDelay”:false,”units”:”hr”,”reset”:”false”,”bytopic”:”all”,”topic”:”topic”,”outputs”:1,”x”:1620,”y”:500,”wires”:[[“9bc5cf63289676e9”,”a23b424ce31ab227”]]},{“id”:”dbe36386e2473459”,”type”:”trigger”,”z”:”66d22c941c2b4f7a”,”name”:”main pump turn ON technical fault solved”,”op1”:”System Status: The main pump is back ON after technical problem is solved!”,”op2”:”0”,”op1type”:”str”,”op2type”:”str”,”duration”:”-12”,”extend”:false,”overrideDelay”:false,”units”:”hr”,”reset”:”false”,”bytopic”:”all”,”topic”:”topic”,”outputs”:1,”x”:1180,”y”:240,”wires”:[[“9bc5cf63289676e9”,”a23b424ce31ab227”]]},{“id”:”48b7513858853470”,”type”:”trigger”,”z”:”66d22c941c2b4f7a”,”name”:”Main pump turn off technical fault”,”op1”:”System Status: The main pump is turned OFF due to some technical problem!”,”op2”:”0”,”op1type”:”str”,”op2type”:”str”,”duration”:”-12”,”extend”:false,”overrideDelay”:false,”units”:”hr”,”reset”:”false”,”bytopic”:”all”,”topic”:”topic”,”outputs”:1,”x”:1180,”y”:320,”wires”:[[“9bc5cf63289676e9”,”a23b424ce31ab227”]]},{“id”:”ae3af01f2e75c7c1”,”type”:”change”,”z”:”66d22c941c2b4f7a”,”name”:”Switch”,”rules”:[{“t”:”set”,”p”:”payload”,”pt”:”msg”,”to”:”true”,”tot”:”bool”}],”action”:”“,”property”:”“,”from”:”“,”to”:”“,”reg”:false,”x”:830,”y”:880,”wires”:[[“48b7513858853470”]]},{“id”:”ef2c1e7cda63d85f”,”type”:”change”,”z”:”66d22c941c2b4f7a”,”name”:”Switch”,”rules”:[{“t”:”set”,”p”:”payload”,”pt”:”msg”,”to”:”true”,”tot”:”bool”}],”action”:”“,”property”:”“,”from”:”“,”to”:”“,”reg”:false,”x”:1270,”y”:540,”wires”:[[“efcaa6991f25e221”]]},{“id”:”d5995736ed3b77f9”,”type”:”influxdb out”,”z”:”66d22c941c2b4f7a”,”influxdb”:”ddfa7dc55332b837”,”name”:”Solar_Current”,”measurement”:”SOLAR_CURRENT”,”precision”:”“,”retentionPolicy”:”“,”database”:”database”,”precisionV18FluxV20”:”ms”,”retentionPolicyV18Flux”:”“,”org”:”organisation”,”bucket”:”bucket”,”x”:780,”y”:60,”wires”:[]},{“id”:”bd5c2393b7f0e08a”,”type”:”influxdb out”,”z”:”66d22c941c2b4f7a”,”influxdb”:”ddfa7dc55332b837”,”name”:”Solar_Voltage”,”measurement”:”SOLAR_VOLTAGE”,”precision”:”“,”retentionPolicy”:”“,”database”:”database”,”precisionV18FluxV20”:”ms”,”retentionPolicyV18Flux”:”“,”org”:”organisation”,”bucket”:”bucket”,”x”:780,”y”:120,”wires”:[]},{“id”:”8721f1d8768e39d7”,”type”:”influxdb out”,”z”:”66d22c941c2b4f7a”,”influxdb”:”ddfa7dc55332b837”,”name”:”Inlet_Pressure”,”measurement”:”INLET_PRESSURE”,”precision”:”“,”retentionPolicy”:”“,”database”:”database”,”precisionV18FluxV20”:”ms”,”retentionPolicyV18Flux”:”“,”org”:”organisation”,”bucket”:”bucket”,”x”:780,”y”:180,”wires”:[]},{“id”:”7c7308e1a2d2e42b”,”type”:”influxdb out”,”z”:”66d22c941c2b4f7a”,”influxdb”:”ddfa7dc55332b837”,”name”:”Inlet_Temperature”,”measurement”:”INLET_TEMPERATURE”,”precision”:”“,”retentionPolicy”:”“,”database”:”database”,”precisionV18FluxV20”:”ms”,”retentionPolicyV18Flux”:”“,”org”:”organisation”,”bucket”:”bucket”,”x”:790,”y”:240,”wires”:[]},{“id”:”5a9cc90c0e745f91”,”type”:”influxdb out”,”z”:”66d22c941c2b4f7a”,”influxdb”:”ddfa7dc55332b837”,”name”:”Pump_Pressure”,”measurement”:”PUMP_PRESSURE”,”precision”:”“,”retentionPolicy”:”“,”database”:”database”,”precisionV18FluxV20”:”ms”,”retentionPolicyV18Flux”:”“,”org”:”organisation”,”bucket”:”bucket”,”x”:780,”y”:300,”wires”:[]},{“id”:”5f631e9dfb48c4b3”,”type”:”influxdb out”,”z”:”66d22c941c2b4f7a”,”influxdb”:”ddfa7dc55332b837”,”name”:”Outlet_Pressure”,”measurement”:”OUTLET_PRESSURE”,”precision”:”“,”retentionPolicy”:”“,”database”:”database”,”precisionV18FluxV20”:”ms”,”retentionPolicyV18Flux”:”“,”org”:”organisation”,”bucket”:”bucket”,”x”:780,”y”:360,”wires”:[]},{“id”:”7d9422173149e539”,”type”:”influxdb out”,”z”:”66d22c941c2b4f7a”,”influxdb”:”ddfa7dc55332b837”,”name”:”Tank_Level”,”measurement”:”TANK_LEVEL”,”precision”:”“,”retentionPolicy”:”“,”database”:”database”,”precisionV18FluxV20”:”ms”,”retentionPolicyV18Flux”:”“,”org”:”organisation”,”bucket”:”bucket”,”x”:770,”y”:420,”wires”:[]},{“id”:”56124fdcb3c89af7”,”type”:”influxdb out”,”z”:”66d22c941c2b4f7a”,”influxdb”:”ddfa7dc55332b837”,”name”:”Pump_Status”,”measurement”:”PUMP_STATUS”,”precision”:”“,”retentionPolicy”:”“,”database”:”database”,”precisionV18FluxV20”:”ms”,”retentionPolicyV18Flux”:”“,”org”:”organisation”,”bucket”:”bucket”,”x”:780,”y”:480,”wires”:[]},{“id”:”9c48f7d63d15a4bb”,”type”:”influxdb out”,”z”:”66d22c941c2b4f7a”,”influxdb”:”ddfa7dc55332b837”,”name”:”System_Status”,”measurement”:”SYSTEM_STATUS”,”precision”:”“,”retentionPolicy”:”“,”database”:”database”,”precisionV18FluxV20”:”ms”,”retentionPolicyV18Flux”:”“,”org”:”organisation”,”bucket”:”bucket”,”x”:780,”y”:540,”wires”:[]},{“id”:”46fd27459ff5a257”,”type”:”ui_group”,”name”:”Inlet Parameters”,”tab”:”2de2fb68707c8cbd”,”order”:1,”disp”:true,”width”:”5”,”collapse”:false,”className”:”“},{“id”:”67e5b6569270f44d”,”type”:”ui_group”,”name”:”Electrical Data”,”tab”:”2de2fb68707c8cbd”,”order”:4,”disp”:true,”width”:”5”,”collapse”:false,”className”:”“},{“id”:”1aa03ee7932a5729”,”type”:”ui_group”,”name”:”System Control”,”tab”:”de854b67da3161d0”,”order”:1,”disp”:true,”width”:”5”,”collapse”:false,”className”:”“},{“id”:”6c6c1fbf4cf20203”,”type”:”arduino-board”,”device”:”/dev/ttyACM0”},{“id”:”7b6e6479fec5ed09”,”type”:”ui_group”,”name”:”Outlet Parameters”,”tab”:”2de2fb68707c8cbd”,”order”:3,”disp”:true,”width”:”5”,”collapse”:false,”className”:”“},{“id”:”28bf606ea75adadd”,”type”:”ui_group”,”name”:”Pump Pressure”,”tab”:”2de2fb68707c8cbd”,”order”:2,”disp”:true,”width”:”5”,”collapse”:false,”className”:”“},{“id”:”84222b1d01e60129”,”type”:”ui_group”,”name”:”Pump Control”,”tab”:”de854b67da3161d0”,”order”:2,”disp”:true,”width”:”5”,”collapse”:false,”className”:”“},{“id”:”3d3554addfd3284e”,”type”:”ui_group”,”name”:”Alerts Management”,”tab”:”de854b67da3161d0”,”order”:3,”disp”:true,”width”:”6”,”collapse”:false,”className”:”“},{“id”:”eabf10c445de3f5a”,”type”:”ui_group”,”name”:”**Latest Update Sent to Community**”,”tab”:”de854b67da3161d0”,”order”:4,”disp”:true,”width”:”5”,”collapse”:false,”className”:”“},{“id”:”ddfa7dc55332b837”,”type”:”influxdb”,”hostname”:”127.0.0.1”,”port”:”8086”,”protocol”:”http”,”database”:”REVERSE_OSMOSIS_DATABASE”,”name”:”“,”usetls”:false,”tls”:”“,”influxdbVersion”:”1.x”,”url”:”http://localhost:8086“,”rejectUnauthorized”:true},{“id”:”2de2fb68707c8cbd”,”type”:”ui_tab”,”name”:”Solar Powered Reverse Osmosis System - Process Parameters”,”icon”:”dashboard”,”order”:2,”disabled”:false,”hidden”:false},{“id”:“de854b67da3161d0”,“type”:”ui_tab”,”name”:”Solar Powered Reverse Osmosis System - Main Control”,”icon”:“fa-fire”,”order”:1,”disabled”:false,”hidden”:false}]
